# The complete chloroplast genomes of *persicaria hydropiper* and *P. pubescens* (polygonaceae)

**DOI:** 10.1080/23802359.2025.2567461

**Published:** 2025-10-08

**Authors:** Yongmei Chen, Yuchun Liu, Jian Li, Qingwen Wei, Jing Wang

**Affiliations:** School of Chemical Engineering, Sichuan University of Science & Engineering, Zigong, China

**Keywords:** Polygonaceae, plastid genome, phylogenetic analysis, persicaria

## Abstract

*Persicaria hydropiper* (L.) Spach and *P. pubescens* (Blume) Hara (Polygonaceae) are widely used as traditional Chinese medicinal plants. In this study, the complete chloroplast (cp) genomes of *P. hydropiper* and *P. pubescens* were sequenced, assembled, and annotated. The cp genome sizes of *P. hydropiper* and *P. pubescens* are 159,054 bp and 159,502 bp, respectively. The cp genomes of *P. hydropiper* and *P. pubescens* each contain a total of 130 genes, including 86 protein-coding, 37 tRNA, and 8 rRNA genes. Phylogenetic analysis reveals that *P. hydropiper* is close to *P. pubescens and P. japonica*, and *Persicaria* is mostly closely related to *Bistorta*.

## Introduction

*Persicaria* (Polygonaceae) is a large genus with about 150 species distributed worldwide, and most of them are used as traditional medicinal plants (Brandbyge 1993; Kim and Donoghue 2008). Two species, *Persicaria hydropiper* (L._Delarbre 1800) and *P. pubescens* (Blume_H.Hara 1941), are the main components of the Chinese traditional patent medicine Jian Qu. Both species are rich in flavonoids, terpenoids and steroids (Liu et al. 2009; Ayaz et al. 2020), exhibiting great potential in the development of pesticides, medicine, veterinary drugs and food additives (Seimandi et al. 2021). However, the lack of genomic information has limited the understanding of its evolutionary relationships and molecular identification (Wolfe and Li 2003; Cooper and Brown 2008). Herein, we report the complete chloroplast (cp) genome sequences of the two species, which can be used for molecular identification of the two medicinal plants and molecular phylogeny studies of the genus *Persicaria.* This work also aims to establish a genomic foundation for species authentication, conservation, and future studies on the genetic diversity and evolution of *P. hydropiper and P. pubescens*.

## Materials and methods

**Figure 1.**
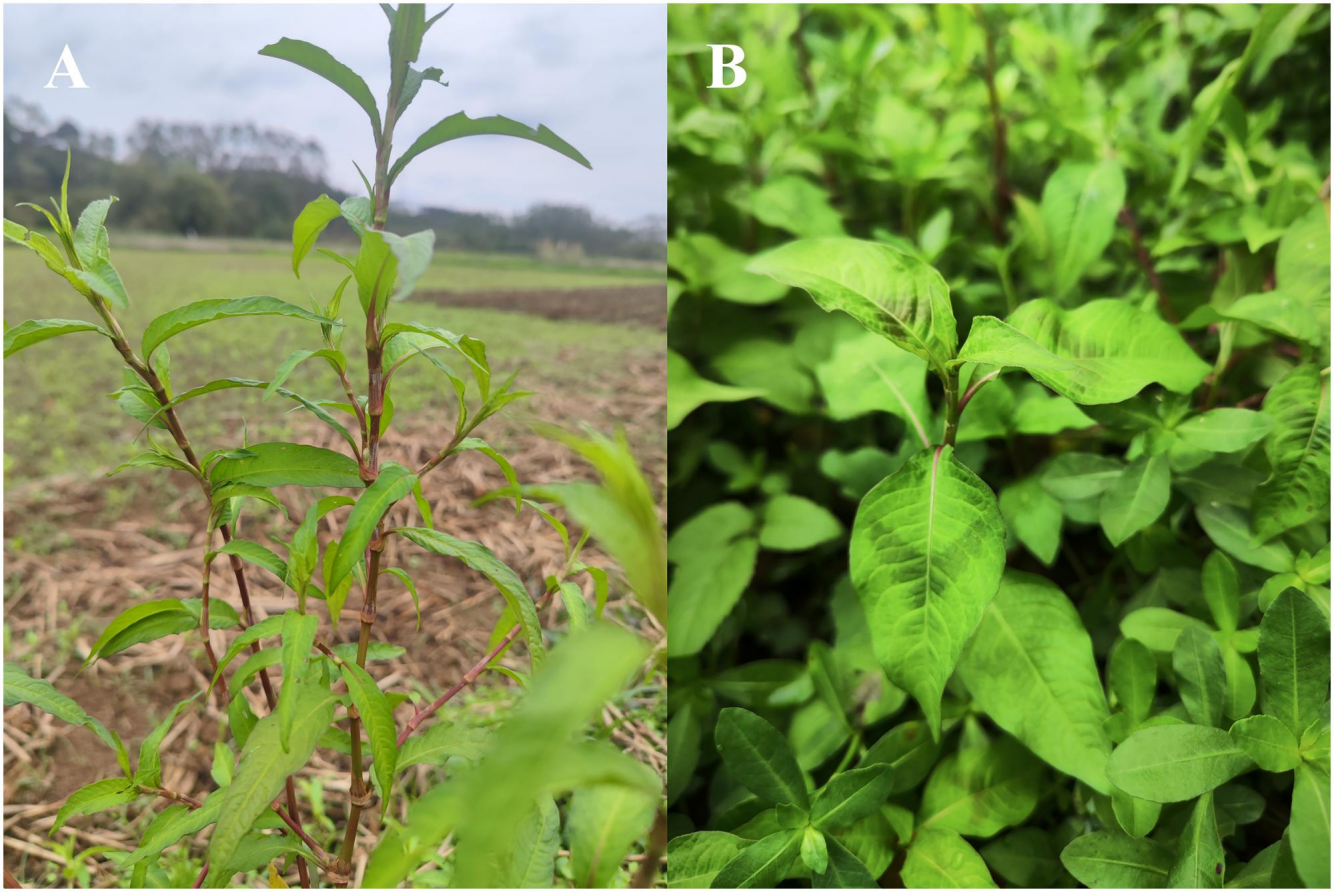
The photos were taken by Yuchun Liu at Sichuan Baisheng Pharmaceutical Co., Ltd. (104.86°E, 29.54°N). (A) *Persicaria hydropiper* is an annual herb (30-80 cm tall) with erect or prostrate stems, lanceolate leaves bearing translucent glands with a pungent taste, slender racemose inflorescences, small greenish-white flowers, and shiny black ovoid achenes. It commonly grows in wetlands and ditches. (B) *Persicaria pubescens* is an annual herb (40-100 cm tall) with erect stems, densely pubescent throughout; leaves lanceolate to ovate-lanceolate, usually hairy; inflorescences dense and erect racemes with pink to reddish flowers; achenes dark brown to black. It often occurs along field margins and moist habitats.

Genomic DNAs were extracted from fresh leaf samples of *P. hydropiper* and *P. pubescens* (Figure 1), which are cultivated and identified by Sichuan Baisheng Pharmaceutical Co., Ltd. (104.86°E, 29.54°N). A specimen was deposited at SUSE (http://www.suse.edu.cn/, Chen Yongmei and chyongm@qq.com) under the voucher number HLL001 and LL001. Genomic shotgun sequencing was performed on the Illumina Hiseq X Ten platform (Nair et al. 2018). About 4 Gb of paired-end 450 bp short reads were generated for each species and then used for assembling their cp genomes with NOVOPlasty (Dierckxsens et al. 2017). Gene annotation was conducted using GeSeq (Tillich et al. 2017). The circular genome map and cis/trans-splicing genes were drawn using CPGView (Liu et al. 2023). Use CPStools to draw the coverage depth map (Huang et al. 2024). Subsequently, the maximum likelihood method was used to conduct a phylogenetic analysis of RAxML (Stamatakis 2014; Ronquist et al. 2012).

## Results

**Figure 2.**
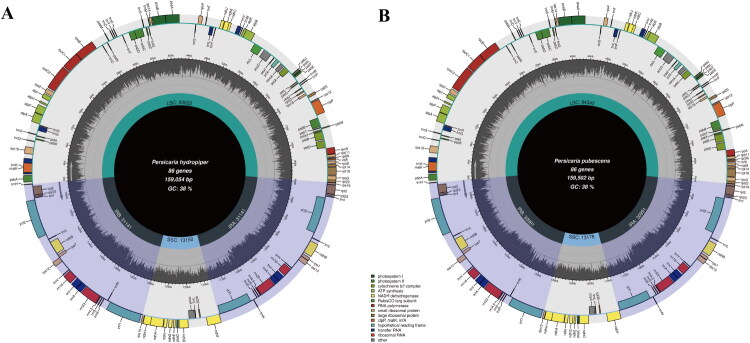
The complete chloroplast genome map of *persicaria hydropiper* (A) and *persicaria pubescens* (B). The cp genome consists of a typical four-region circular molecule with a large single-copy (LSC) region, a small single-copy (SSC) region, and a pair of inverted repeats (IRA and IRB) regions. The genes transcribed in clockwise and counterclockwise directions are shown on the inner and outer sides of the circle, respectively.

**Figure 3.**
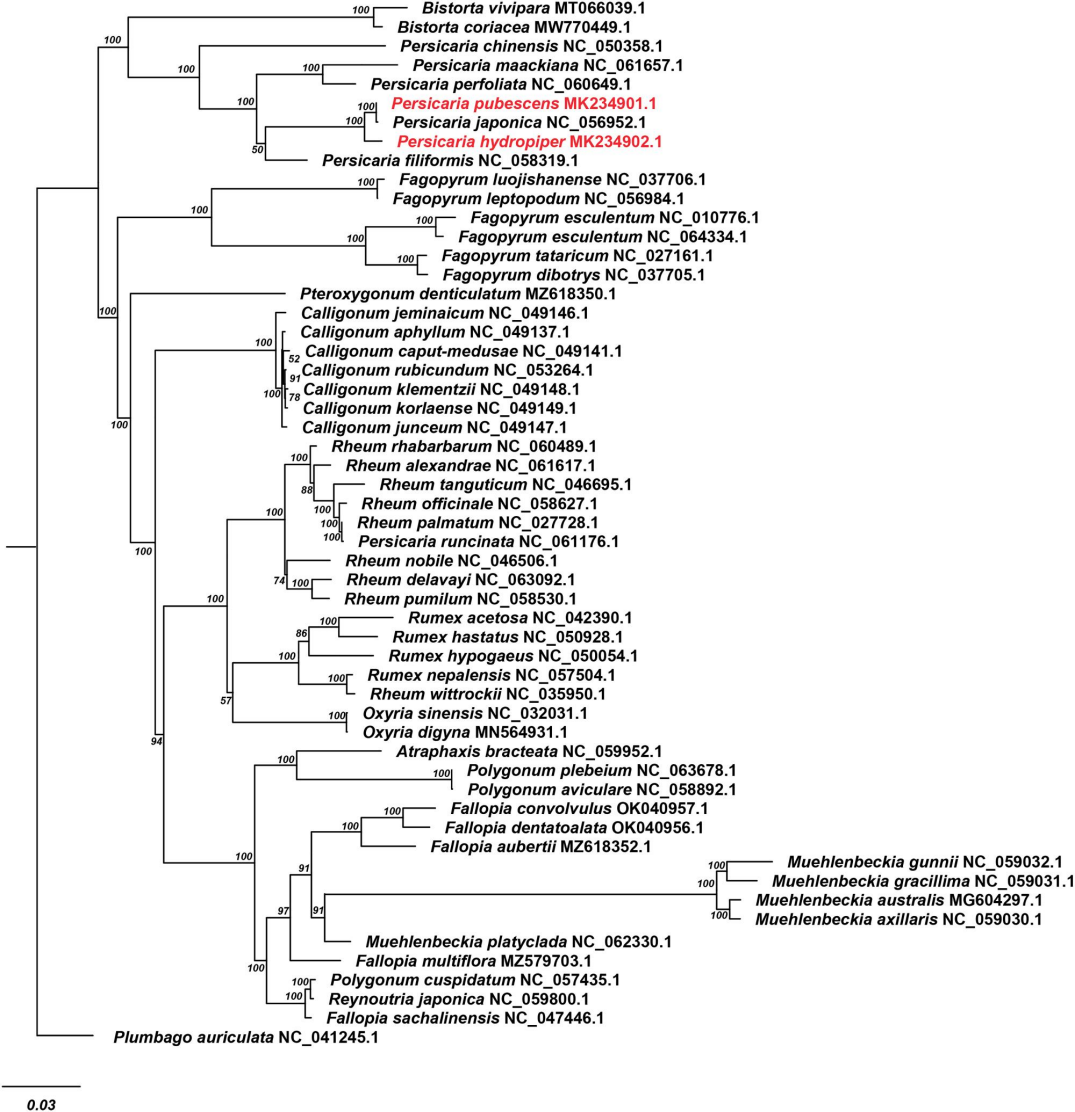
Maximum-likelihood tree of 54 taxa of polygonaceae based on their complete chloroplast proteome sequences, with *Plumbago auriculata* from Plumbagineae as the outgroup. Bootstrap values are indicated at each node. Phylogenetic analysis was conducted based on complete chloroplast genome sequences. The species names, GenBank accession numbers, and corresponding references are provided in Supplementary Table 1 at the end of the supplementary document.

The complete cp genome sizes of *P. hydropiper* and *P. pubescens* are 159,054 bp and 159,502 bp, respectively. The cp genomes of *P. hydropiper* and *P. pubescens* each have a total of 130 genes, including 86 protein-coding, 37 transfer RNA (tRNA), and 8 ribosomal RNA (rRNA) genes (Figure 2). The overall depths of coverage for the assembled genomes are illustrated in FigureS 1 and FigureS 2. The genome of *P. hydropiper* has 23 genes with introns, of which three genes have two introns (*clpP,* and two *rps12*), and 20 genes have one intron (*rps16*、*atpF*、*rpoC1*、*petB*、*petD*、two *ndhB*、*ndhA*、*rpl16*、two *trnA-UGC*、*trnA-GUG*、two *trnA-UCC*、two *trnL-GAU*、*trnK-UUU*、*trnL-UAA*、*trnG-UCC*、and *trnV-UAC*). The genome contained 10 cis-splicing genes and one trans-splicing gene (FigureS 3). The genome of *P. pubescens* has 18 genes with introns, of which three genes have two introns (*clpP*, and two *rps12*), and 15genes have one intron (*rps16*、*atpF*、*rpoC1*、*petB*、*petD*、two *ndhB*、*ndhA*、*rpl16*、two *trnA-UGC*、*trnK-UUU*、*trnL-UAA*、*trnG-UCC*、and *trnV-UAC*). The genome contained 10 cis-splicing genes and one trans-splicing gene (Figure S4).

Phylogenetic analysis was performed using the maximum likelihood method in RAxML (Stamatakis 2014), based on the complete cp proteome sequences of 54 taxa representing thirteen genera from Polygonaceae and an outgroup species *Plumbago auriculata* from Plumbagineae. The maximum-likelihood tree reveals that *P. hydropiper* is close to *P. pubescens and P. japonica*, and *Persicaria* is mostly closely related to *Bistorta* (Figure 3).

## Discussion and conclusions

In conclusion, the present study successfully sequenced, assembled, and annotated the complete chloroplast (cp) genomes of *Persicaria hydropiper* and *P. pubescens*, providing valuable genomic resources for these species. The cp genome sizes of *P. hydropiper* and *P. pubescens* were determined to be 159,054 bp and 159,502 bp, respectively, which are typical for angiosperms and consistent with the size range of other cp genomes sequenced to date (Daniell et al. 2016). Both genomes contain a similar set of 130 genes, comprising 86 protein-coding genes, 37 tRNA genes, and 8 rRNA genes, highlighting the conservation of gene content among these species. While previous chloroplast studies on Persicaria remain limited, our findings contribute essential genetic information at the organellar level, serving as a valuable reference for future taxonomic, phylogenetic, and conservation efforts (Zhou et al. 2024; Zeng et al. 2025). Nevertheless, this research was restricted to the chloroplast genome and did not integrate nuclear or transcriptomic data, which may limit the comprehensive interpretation of interspecific variation (Schultheis et al. 2014). The phylogenetic analysis conducted in this study revealed that *P. hydropiper* is closely related to *P. pubescens* and *P. japonica*, which is in agreement with previous taxonomic classifications and provides molecular evidence supporting their genetic proximity (Takabe et al. 2018; Choi et al. 2022). Based on our findings, future studies should incorporate broader population sampling and integrate multi-omics approaches to further investigate the genetic diversity, biosynthetic pathways of active compounds, and adaptive evolution of Persicaria. Such efforts will provide deeper insights into its medicinal potential and support the sustainable utilization of this traditional herb.

## Supplementary Material

Supplemental_material (clean).docx

## Data Availability

The genome sequence data that support the findings of this study are openly available in the NCBI GenBank (http://www.ncbi.nlm.nih.gov) under accession numbers MK234901.1-MK234902.1. The associated BioProject, SRA, and Bio-Sample numbers are PRJNA827105, SRR18791638-SRR18791639, and SAMN27596958-SAMN27596959 respectively.
