# Supplementary material for: The complete chloroplast genomes of persicaria hydropiper and P. pubescens (polygonaceae)
Source: Mitochondrial DNA B Resour. 2025 Oct 8;10(11):1008–11. doi: 10.1080/23802359.2025.2567461 (PMC12509295; doi:10.1080/23802359.2025.2567461)
Supplement: Supplemental_material (clean).docx [file TMDN_A_2567461_SM2767.docx]

The complete chloroplast genomes of *Persicaria hydropiper* and *P. pubescens* (Polygonaceae)

ORCID

Yongmei Chen：0009-0007-0680-1801

Yuchun Liu：0009-0007-5119-7973

Jian Li：0009-0008-6724-9931

Qingwen Wei：0009-0003-2260-3515

Jing Wang：0009-0000-2731-4120


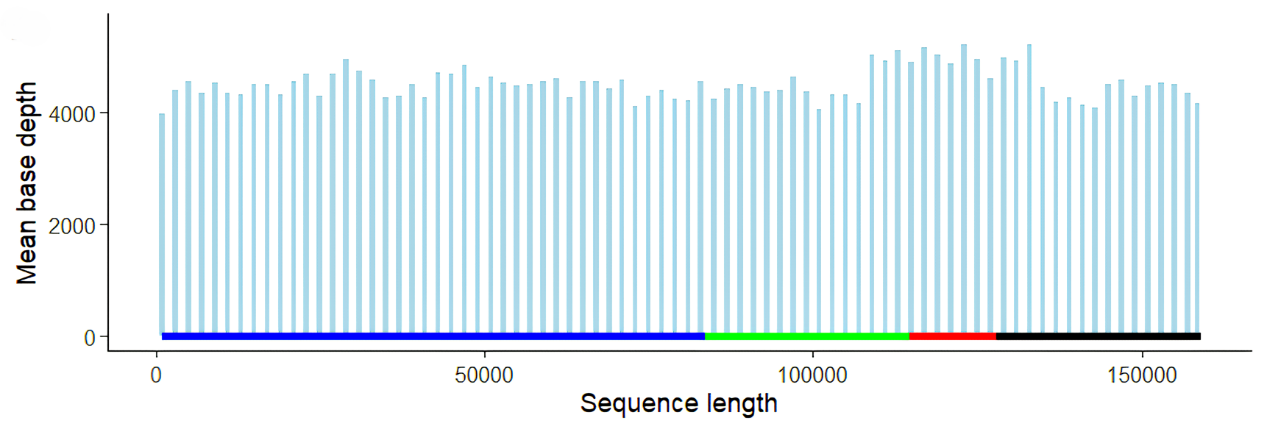


Figure S1. Coverage depth figure of the *Persicaria hydropiper* chloroplast genome.The X and Y axes represent the nucleotide position of the *P.hydropiper* and the corresponding coverage depth, respectively.


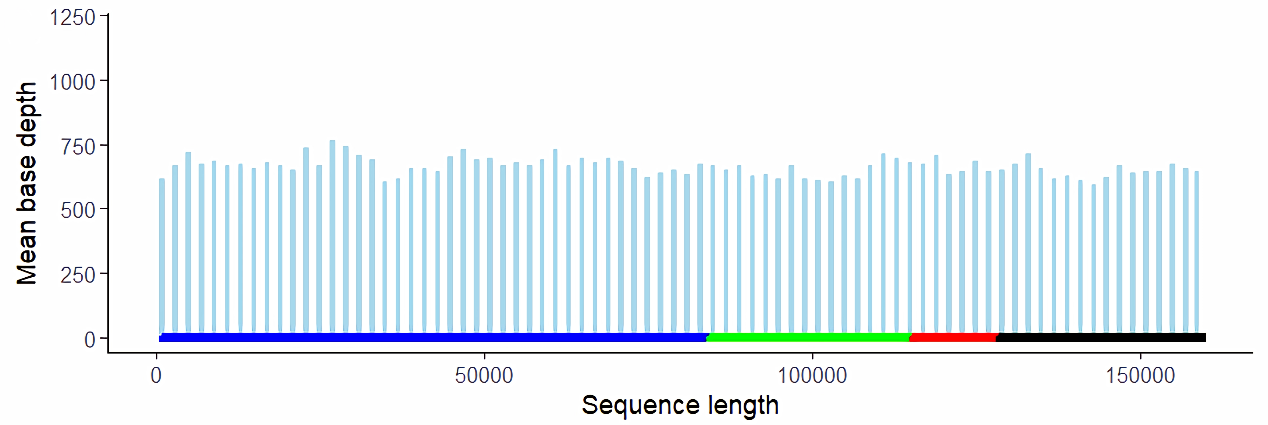


Figure S2. Coverage depth figure of the *Persicaria pubescens* chloroplast genome. The X and Y axes represent the nucleotide position of the *P. pubescens* and the corresponding coverage depth, respectively.


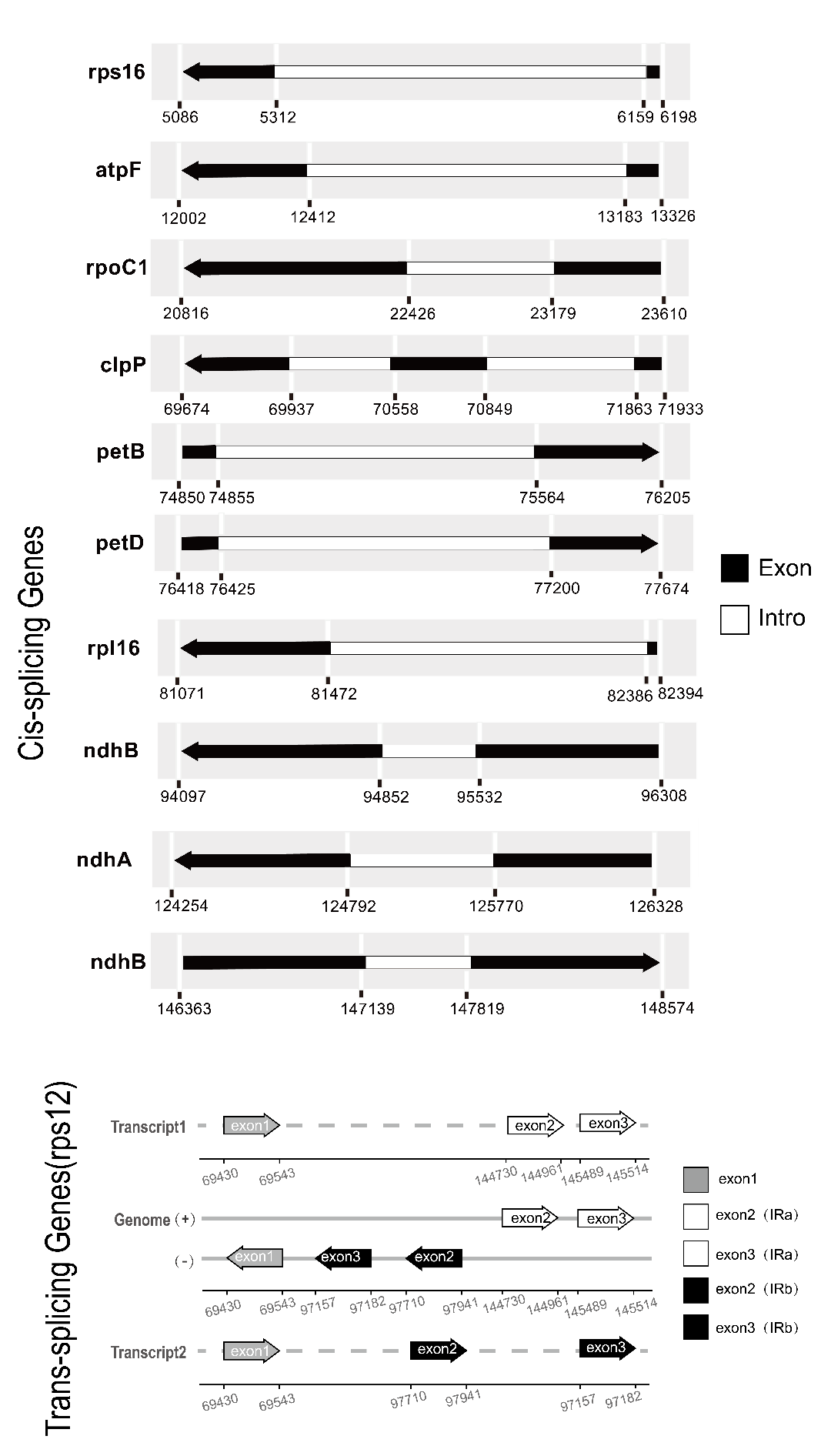


Figure S3. The schematic map of the cis-splicing (*rps16*, *atpF*, *rpoC1*, *clpP*, *petB,* *petD*, *rpl16*, *ndhB* and *ndhA*) and the trans-splicing (*rps12*) genes in the *Persicaria hydropiper* chloroplast genome.


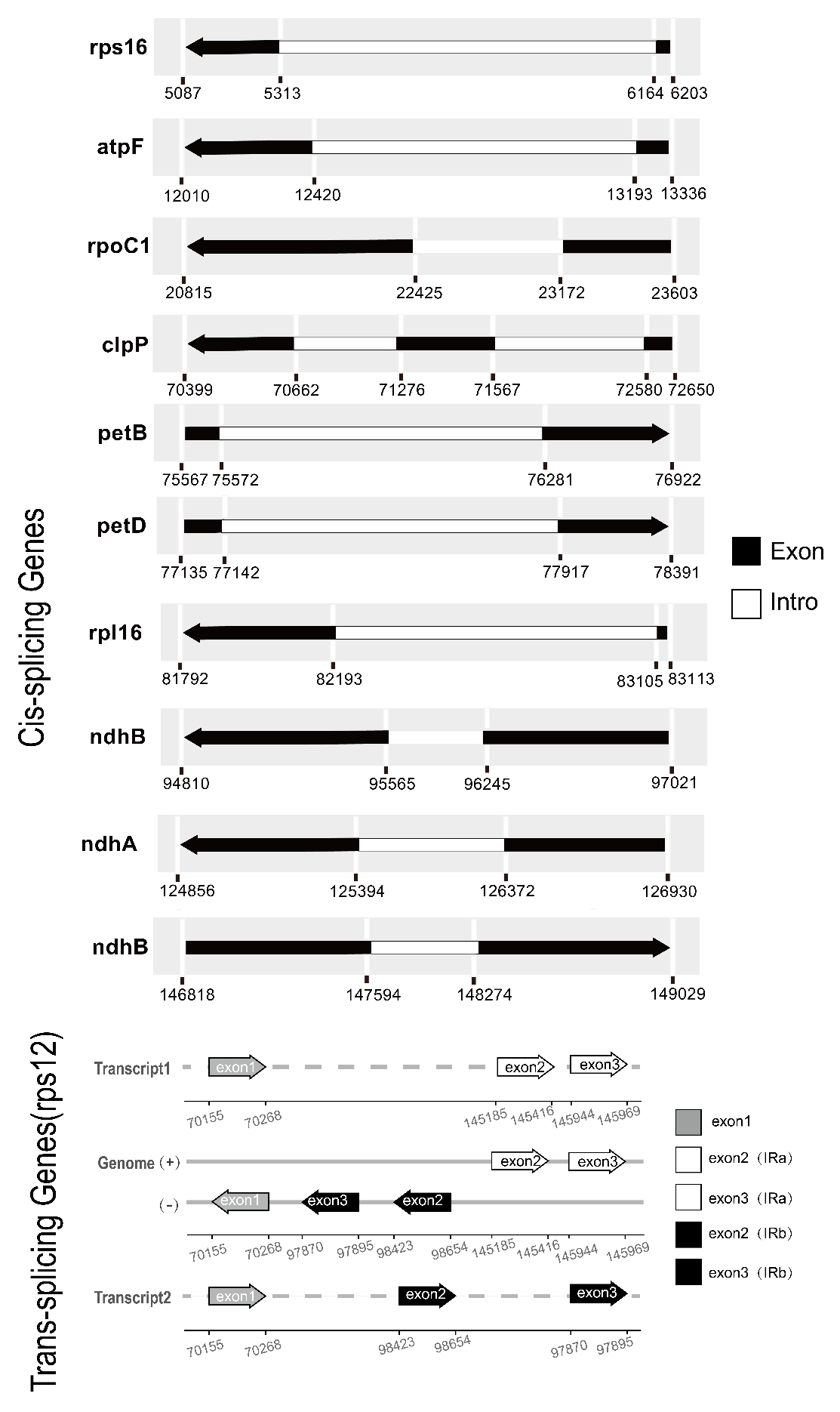


Figure S4. The schematic map of the cis-splicing (*rps16*, *atpF*, *rpoC1*, *clpP*, *petB*, *petD*, *rpl16*, *ndhB* and *ndhA*) and the trans-splicing (*rps12*) genes in the *Persicaria pubescens* chloroplast genome.

Supplementary Table S1. List of all 55 species, GenBank accession numbers and references for sequences used to construct phylogenetic trees (Figure 3).

| **Species** | **Accession number** | **References** |
| --- | --- | --- |
| *Bistorta vivipara*  *Bistorta coriacea*  *Persicaria chinensis*  *Persicaria maackiana*  *Persicaria perfoliata*  *Persicaria pubescens*  *Persicaria japonica*  *Persicaria hydropipe*  *Persicaria filiformis*  *Fagopyrum luojishanense*  *Fagopyrum leptopodum*  *Fagopyrum esculentum*  *Fagopyrum esculentum*  *Fagopyrum tataricum*  *Fagopyrum dibotrys*  *Pteroxygonum denticulatum*  *Calligonum jeminaicum*  *Calligonum aphyllum*  *Calligonum caputmedusae*  *Calligonum rubicundum*  *Calligonum klementzii*  *Calligonum korlaense*  *Calligonum junceum*  *Rheum rhabarbarum*  *Rheum alexandrae*  *Rheum tanguticum*  *Rheum officinale*  *Rheum palmatum*  *Persicaria runcinata*  *Rheum nobile*  *Rheum delavayi*  *Rheum pumilum*  *Rumex acetosa*  *Rumex hastatus*  *Rumex hypogaeus*  *Rumex nepalensis*  *Rheum wittrockii*  *Oxyria sinensis*  *Oxyria digyna*  *Atraphaxis bracteata*  *Polygonum plebeium*  *Polygonum aviculare*  *Fallopia convolvulus*  *Fallopia dentatoalata*  *Fallopia aubertii*  *Muehlenbeckia gunnii*  *Muehlenbeckia gracillima*  *Muehlenbeckia australis*  *Muehlenbeckia axillaris*  *Muehlenbeckia platyclada*  *Fallopia multiflora*  *Polygonum cuspidatum*  *Reynoutria japonica*  *Fallopia sachalinensis*  *Plumbago auriculata* | MT066039.1  MW770449.1  NC_050358.1  NC_061657.1  NC_060649.1  MK234901.1  NC_056952.1  MK234902.1  NC_058319.1  NC_037706.1  NC_056984.1  NC_010776.1  NC_064334.1  NC_027161.1  NC_037705.1  MZ618350.1  NC_049146.1  NC_049137.1  NC_049141.1  NC_053264.1  NC_049148.1  NC_049149.1  NC_049147.1  NC_060489.1  NC_061617.1  NC_046695.1  NC_058627.1  NC_058627.1  NC_061176.1  NC_046506.1  NC_063092.1  NC_058530.1  NC_042390.1  NC_050928.1  NC_050054.1  NC_057504.1  NC_035950.1  NC_032031.1  MN564931.1  NC_059952.1  NC_063678.1  NC_058892.1  OK040957.1  OK040956.1  MZ618352.1  NC_059032.1  NC_059031.1  MG604297.1  NC_059030.1  NC_062330.1  MZ579703.1  NC_057435.1  NC_059800.1  NC_047446.1  NC_041245.1 | Direct Submission  Direct Submission  Yu et al. 2020  Direct Submission  Direct Submission  This study  Direct Submission  This study  Direct Submission  Wang et al. 2017  Direct Submission  Logacheva et al. 2008  Direct Submission  Cho et al. 2015  Wang et al. 2017  Direct Submission  Direct Submission  Song et al. 2020  Direct Submission  Song et al. 2020  Direct Submission  Direct Submission  Direct Submission  Direct Submission  Direct Submission  Huo et al. 2019  Li et al. 2019  Li et al. 2019  Direct Submission  Direct Submission  Direct Submission  Li et al. 2020  Gui et al. 2018  Yang et al. 2020  Choi et al. 2020  Wu et al. 2020  Dagarova et al. 2017  Direct Submission  Direct Submission  Direct Submission  Direct Submission  Direct Submission  Direct Submission  Direct Submission  Direct Submission  Maurin et al. 2022  Maurin et al. 2022  Direct Submission  Maurin et al. 2022  Direct Submission  Direct Submission  Direct Submission  Direct Submission  Raman et al. 2019  Yao et al. 2019 |

**GenBank References**

Yu B, Liu J, Liu X, et al. 2020. The complete chloroplast genome sequence of *Polygonum chinense* L[J]. Mitochondrial DNA Part B, 5(3): 2139-2140. doi: org/10.1080/23802359.2019.1693931.

Wang C L, Ding M Q, Zou C Y, et al. 2017. Comparative analysis of four buckwheat species based on morphology and complete chloroplast genome sequences[J]. Scientific Reports, 7(1): 6514. doi: 10.1038/s41598-017-06638-6.

Logacheva M D, Samigullin T H, Dhingra A, et al. 2008. Comparative chloroplast genomics and phylogenetics of *Fagopyrum esculentum* ssp. *Ancestrale*-a wild ancestor of cultivated buckwheat[J]. BMC Plant Biology, 8(1): 59. doi: 10.1186/1471-2229-8-59.

Cho K S, Yun B K, Yoon Y H, et al. 2015. Complete chloroplast genome sequence of tartary buckwheat (*Fagopyrum tataricum*) and comparative analysis with common buckwheat (*F. esculentum*)[J]. PLoS One, 10(5): e0125332. doi: org/10.1371/journal.pone.0125332.

Song F, Li T, Burgess K S, et al. 2020. Complete plastome sequencing resolves taxonomic relationships among species of *Calligonum* L.(Polygonaceae) in China[J]. BMC Plant Biology, 20(1): 261. 61. doi: org/10.1186/s12870-020-02466-5.

Huo X, Wei H, Gao J, et al. 2019. The complete chloroplast genome sequence of *Rheum tanguticum*, an endangered Chinese medicinal plant (Polygonaceae)[J]. Mitochondrial DNA Part B, 4(2): 4055-4056. doi: org/10.1080/23802359.2019.1688722.

Li Y, Li H, Hei X, et al. 2019. Characterization of the complete chloroplast genome of medicinal plant *Rheum officinale* (Polygonaceae)[J]. Mitochondrial DNA Part B, 4(2): 2144-2145. doi: org/10.1080/23802359.2019.1623102.

Li R, Zhang X, Wang J, et al. 2020. Characterization of the complete chloroplast genome of traditional Tibetan herb, *Rheum Pumilum* Maxim.(Polygonaceae)[J]. Mitochondrial DNA Part B, 5(1): 133-135. doi: org/10.1080/23802359.2019.1698344.

Gui L, Jiang S, Wang H, et al. 2018. Characterization of the complete chloroplast genome of sorrel (*Rumex acetosa*)[J]. Mitochondrial DNA Part B, 3(2): 902-904. doi: org/10.1080/23802359.2018.1501297.

Yang K, Shang M, Jiang Y, et al. 2020. The complete chloroplast genome of *Rumex hastatus* D. Don and its phylogenetic analysis[J]. Mitochondrial DNA Part B, 5(2): 1681-1682. doi: org/10.1080/23802359.2020.1746705.

Choi K S, Lee W H, Park S J. 2020. The complete chloroplast genome of *Emex australis* (Polygonaceae)[J]. Mitochondrial DNA Part B, 5(2): 1431-1432. doi: org/10.1080/23802359.2020.1736962.

Wu C X, Zhai C C, Fan S J. 2020. Characterization of the complete chloroplast genome of *Rumex nepalensis* (Polygonaceae)[J]. Mitochondrial DNA Part B, 5(3): 2458-2459. doi: org/10.1080/23802359.2020.1778568.

Dagarova S S, Sitpayeva G T, Pak J H, et al. 2017. The complete plastid genome sequence of *Rheum wittrockii* (Polygonaceae), endangered species of Kazakhstan[J]. Mitochondrial DNA Part B, 2(2): 516-517. doi: org/10.1080/23802359.2017.1361359.

Maurin K J L, Smissen R D, Lusk C H. 2022. A dated phylogeny shows Plio‐Pleistocene climates spurred evolution of antibrowsing defences in the New Zealand flora[J]. New Phytologist, 233(1): 546-554. doi: org/10.1111/nph.17766.

Raman G, Park K T, Nam G H, et al. 2019. Characterization of the complete chloroplast genome sequence of the giant knotweed, *Fallopia sachalinensis* from the volcanic island Dokdo, Republic of Korea[J]. Mitochondrial DNA Part B, 4(2): 2972-2973. doi: org/10.1080/23802359.2019.1663769.

Yao G, Jin J J, Li H T, et al. 2019. Plastid phylogenomic insights into the evolution of Caryophyllales[J]. Molecular Phylogenetics and Evolution, 134: 74-86. doi: org/10.1016/j.ympev.2018.12.023.
